# Deep clinical, genetic, and serum biomarker profiling indicates glial and neuronal pathology in primary brain calcification

**DOI:** 10.1093/braincomms/fcaf388

**Published:** 2025-10-07

**Authors:** Janine Schwahn, Sophie Hebestreit, Olivia Kosche, Petra Steinacker, Vesile Sandikci, Isabel Winzer, Jasper Hesebeck-Brinckmann, Franziska Bachhuber, Ivan Valkadinov, Stefanie Nittka, Lukas Mesin, Max Brauner, Christine von Arnim, Nandhini Santhanam, Marvin Spreyer, Robert Sackmaier, Antje Knehr, Paula Barthel, Oliver Bähr, Herbert Gruber, Sabine Stallforth, Martin Regensburger, Jürgen Winkler, Rüstem Yilmaz, Michael Neumaier, Hayrettin Tumani, Mate E Maros, Holger Wenz, David Brenner, Markus Otto, Anne Ebert, Julian Conrad, Jochen H Weishaupt

**Affiliations:** Division of Neurodegeneration, Department of Neurology, Mannheim Center for Translational Neurosciences, Medical Faculty Mannheim, Heidelberg University, Mannheim 68167, Germany; Division of Neurodegeneration, Department of Neurology, Mannheim Center for Translational Neurosciences, Medical Faculty Mannheim, Heidelberg University, Mannheim 68167, Germany; Division of Neurodegeneration, Department of Neurology, Mannheim Center for Translational Neurosciences, Medical Faculty Mannheim, Heidelberg University, Mannheim 68167, Germany; Department of Neurology, Martin-Luther-University of Halle-Wittenberg, Halle (Saale) 06120, Germany; Department of Neurology, Medical Faculty Mannheim, Heidelberg University, Mannheim 68167, Germany; Division of Neurodegeneration, Department of Neurology, Mannheim Center for Translational Neurosciences, Medical Faculty Mannheim, Heidelberg University, Mannheim 68167, Germany; Division of Neurodegeneration, Department of Neurology, Mannheim Center for Translational Neurosciences, Medical Faculty Mannheim, Heidelberg University, Mannheim 68167, Germany; Department of Neurology, University of Ulm, Ulm 89081, Germany; Department of Neurology, University of Ulm, Ulm 89081, Germany; Division of Neurodegeneration, Department of Neurology, Mannheim Center for Translational Neurosciences, Medical Faculty Mannheim, Heidelberg University, Mannheim 68167, Germany; Institute for Clinical Chemistry, Medical Faculty Mannheim, Heidelberg University, Mannheim 68167, Germany; Division of Neurodegeneration, Department of Neurology, Mannheim Center for Translational Neurosciences, Medical Faculty Mannheim, Heidelberg University, Mannheim 68167, Germany; Division of Neurodegeneration, Department of Neurology, Mannheim Center for Translational Neurosciences, Medical Faculty Mannheim, Heidelberg University, Mannheim 68167, Germany; Department of Geriatrics, University of Göttingen Medical Center, Göttingen 37075, Germany; Department of Biomedical Informatics, Medical Faculty Mannheim, Heidelberg University, Mannheim 68167, Germany; Division of Neurodegeneration, Department of Neurology, Mannheim Center for Translational Neurosciences, Medical Faculty Mannheim, Heidelberg University, Mannheim 68167, Germany; Department of Neurology, Elisabethinen Clinic, Graz 8020, Austria; Division of Neurodegeneration, Department of Neurology, Mannheim Center for Translational Neurosciences, Medical Faculty Mannheim, Heidelberg University, Mannheim 68167, Germany; Department of Neurology, University of Ulm, Ulm 89081, Germany; Division of Neurodegeneration, Department of Neurology, Mannheim Center for Translational Neurosciences, Medical Faculty Mannheim, Heidelberg University, Mannheim 68167, Germany; Department of Neurology, Aschaffenburg-Alzenau Clinic, Aschaffenburg 63739, Germany; Department of Neurology, Aschaffenburg-Alzenau Clinic, Aschaffenburg 63739, Germany; Department of Molecular Neurology, University Hospital Erlangen, Erlangen 91054, Germany; Department of Molecular Neurology, University Hospital Erlangen, Erlangen 91054, Germany; Department of Molecular Neurology, University Hospital Erlangen, Erlangen 91054, Germany; Division of Neurodegeneration, Department of Neurology, Mannheim Center for Translational Neurosciences, Medical Faculty Mannheim, Heidelberg University, Mannheim 68167, Germany; Institute for Clinical Chemistry, Medical Faculty Mannheim, Heidelberg University, Mannheim 68167, Germany; Department of Neurology, University of Ulm, Ulm 89081, Germany; Department of Biomedical Informatics, Medical Faculty Mannheim, Heidelberg University, Mannheim 68167, Germany; Department of Neuroradiology, Medical Faculty Mannheim, Heidelberg University, Mannheim 68167, Germany; Department of Neuroradiology, Medical Faculty Mannheim, Heidelberg University, Mannheim 68167, Germany; Division of Neurodegeneration, Department of Neurology, Mannheim Center for Translational Neurosciences, Medical Faculty Mannheim, Heidelberg University, Mannheim 68167, Germany; Department of Neurology, University of Ulm, Ulm 89081, Germany; Department of Neurology, Martin-Luther-University of Halle-Wittenberg, Halle (Saale) 06120, Germany; Department of Neurology, Medical Faculty Mannheim, Heidelberg University, Mannheim 68167, Germany; Division of Neurodegeneration, Department of Neurology, Mannheim Center for Translational Neurosciences, Medical Faculty Mannheim, Heidelberg University, Mannheim 68167, Germany; Division of Neurodegeneration, Department of Neurology, Mannheim Center for Translational Neurosciences, Medical Faculty Mannheim, Heidelberg University, Mannheim 68167, Germany; Department of Neurology, University of Ulm, Ulm 89081, Germany

**Keywords:** primary brain calcification, basal ganglia calcification, biomarker, neurogenetics, movement disorder

## Abstract

Primary brain calcification (primary familial brain calcification in inherited cases) is an often-genetic condition characterized by symmetrical brain calcifications and neuropsychiatric symptoms. The calcifications can also occur without overt clinical symptoms. Identifying laboratory biomarkers in primary brain calcification and their association with imaging, genetic, and clinical data will be crucial for a deeper understanding of primary brain calcification causation and progression and the planning of therapeutic trials. The serum biomarkers for neuronal degeneration (phosphorylated tau, neuron-specific enolase, neurofilament light- and heavy chain) and glial activation (glial fibrillary acidic protein, S100 calcium-binding protein B) were measured in 101 probands (41 controls and 60 probands with primary brain calcification). The deep phenotyping protocol of the German Fahr-NET register included neurological and neuropsychological examination, routine laboratory workup, and whole exome sequencing. We also performed and analyzed 45 cranial CT scans using the total calcification score. While mild pallidal calcifications were observed early in young, asymptomatic primary brain calcification mutation carriers, individuals with calcifications extending beyond the pallidum were symptomatic. Individuals with primary brain calcification had elevated serum glial fibrillary acidic protein and neurofilament light chain levels. Serum biomarkers correlated with both the extent of calcifications and the clinical impairment. Elevated parathyroid hormone levels distinguished the primary brain calcification group without identified mutation from both genetic primary brain calcification and control groups. Our results define a practical imaging cut-off indicating the presence of primary brain calcification symptoms. Elevated parathyroid hormone levels in primary brain calcification without identified mutation, but not in primary brain calcification with a monogenic cause, suggest abortive calcium regulation defects as a pathogenic factor specifically for primary brain calcification without identified mutation. Elevated glial fibrillary acidic protein and neurofilament concentrations in individuals with primary brain calcification indicate early, chronic astrocytosis and neuronal impairment, respectively. The significant association of neurofilament light chain with clinical scores and brain imaging results will be relevant for future therapeutic studies.

## Teaser

Schwahn *et al*. report that individuals with primary brain calcification had elevated serum glial fibrillary acidic protein and neurofilament light chain levels indicating early, chronic astrocytosis and neuronal impairment. The significant association of neurofilament light chain with clinical scores and brain imaging results will be relevant for future therapeutic studies.

## Graphical Abstract

**Graphical Abstract:**
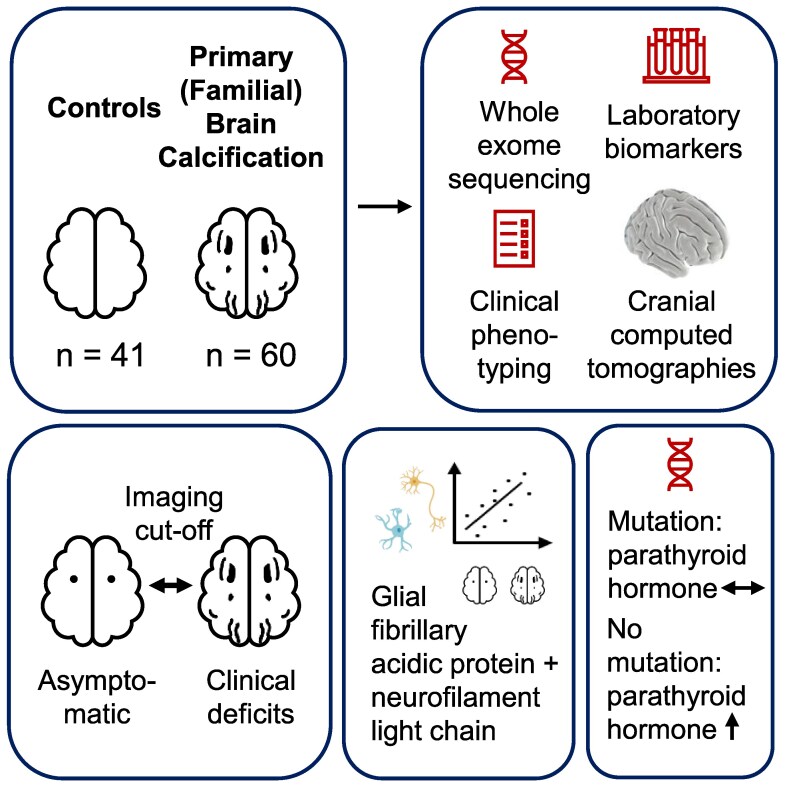

## Introduction

Primary brain calcification (PBC) is characterized by symmetric calcifications in adult individuals’ basal ganglia, thalamus, cerebellum, cerebral cortex, or brain stem. PBC can either occur without clinical symptoms or lead to a broad range of both neurological and neuropsychological deficits, such as movement disorders, bulbar symptoms, cognitive deficits, or psychiatric symptoms, including depression or anxiety.^1^ Calcifications without symptoms are increasingly observed with higher age.^2-4^ Considering also minor and small punctuated deposits in the basal ganglia, a substantial proportion of elderly individuals appear to have asymptomatic calcifications.^3^

Besides other designations [idiopathic basal ganglia calcification (IBGC)], the term primary familial brain calcification (PFBC) has been coined.^1,2,5^ However, a considerable proportion of patients who carry a mutation causing brain calcifications report a negative family history.^2,5^ Henceforth, we will use the more general term PBC when a secondary cause, such as infections, mitochondrial disease, or focal brain lesion, has been excluded. In contrast, PFBC refers to a person with a positive family history of PBC.

In recent years, PBC-causing genetic mutations have been identified in the genes *SLC20A2, PDGFRB, PDGFB,* and *XPR1* linked to autosomal-dominant inheritance. Moreover, *MYORG, JAM2,* and *NAA60* mutations can cause autosomal-recessively inherited PBC.^6-12^ At the time of last examination, 30–42% of individuals with a pathogenic PBC mutation appear asymptomatic and incomplete or age-dependent penetrance seems typical for PBC mutations.^13-15^

The known PBC genes are predominantly expressed in cells of the neurovascular unit that regulate transmembrane ion transport and blood-brain barrier (BBB) integrity, such as astrocytes, endothelial cells and pericytes.^16^ Alterations of the BBB may thus be a common pathogenetic denominator of PBC.^17,18^ Glial changes, such as activated astrocytes and microglia, surround the calcification nodules observed in PBC.^16,19-22^ Differentiation of astrocytes to osteoclast-like cells and microglia activation have been suggested in animal models.^20^ However, autopsy tissue from PBC patients and neuropathological studies on PBC are scarce, and biomarker-based data that would allow correlation of neuronal or glial alterations with clinical deficits and imaging abnormalities during the patients’ lifetime are lacking.

Overall, the relationship between genetic, often familial, symptomatic P(F)BC patients and the more frequently observed sporadic calcifications, usually milder and asymptomatic, have remained largely unclear. A clear imaging-based cut-off separating clinically symptomatic and asymptomatic PBC, independent of the familial and genetic backgrounds, has not been established. Furthermore, laboratory biomarkers for PBC to gain insight into the disease activity in live patients are not available at all. Here, we present data from the German Fahr-NET PBC register and biobank. We analyze the association between the extent of brain calcifications, clinical deficits, genetic status, and laboratory biomarkers that could serve as in vivo indicators for disease activity and cellular pathology. Respective laboratory markers could also serve as readout parameters for future therapeutic trials in the PBC field.

## Materials and methods

### Proband recruiting

Probands were recruited at the neurological clinic of the University Medical Center Mannheim in Germany from June 2021 to February 2024. Sixty probands with brain calcifications were locally referred to the Division for Neurodegeneration (*n* = 52) or from other neurological clinics (*n* = 8). These external clinics include the University Hospital Ulm (*n* = 3), University Hospital Erlangen (*n* = 1), Klinikum Aschaffenburg-Alzenau (*n* = 1), the University Hospital Gießen and Marburg (*n* = 1), and the Elisabethinen Clinic in Graz (*n* = 2). The probands showed a mean age of 56.75 years ±15.36 and a gender distribution of 30/30 (male/female). Inclusion criteria for final data analysis consisted of the following: (i) presence of bilateral calcification of brain tissue observed in cranial CT (cCT) or cranial MRI (cMRI), (ii) normal calcium and phosphate metabolism, and (iv) exclusion of further secondary causes such as traumata or infections. All subjects provided their informed written consent to participate in the registry study ‘Fahr-Net’, granting permission for the use of their data, as well as for whole exome sequencing (WES) and genetic analysis in a scientific context without result communication. Furthermore, 41 controls were included in the study. The mean age of all control probands was 55.94 years ±12.99 with a gender distribution of 17/24 (male/female). 24 (58.5%) of them were healthy controls with a mean age of 55.40 years ±10.85 and a gender distribution of 11/13 (male/female) and 17 (41.5%) were disease controls with a mean age of 56.71 years ±15.85 and a gender distribution of 6/11 (male/female). Further details of disease controls including their diagnoses are also described in Supplementary Table 1. Healthy controls were spouses of patients or were recruited via leaflet advertisements in the outpatient clinic at Mannheim University Hospital. The study was approved by the Medical Ethics Commission II of the Medical Faculty Mannheim, University of Heidelberg (approval no.: 2021-529-MA) and the local participating sites.

### Standard protocol

Subjects with PBC underwent comprehensive neurological examinations and detailed medical history assessments. Moreover, the Scale for the Assessment and Rating of Ataxia (SARA) and the Parkinson`s Disease Questionnaire (PDQ-39), as well as parts I to III of the Unified Parkinson`s Disease Rating Scale (UPDRS) and the Barthel Index were collected. The Montreal Cognitive Assessment (MOCA), Beck Depression Inventory-II (BDI-II), and a behavioral observation during the anamnestic interview and neuropsychological assessment were performed to estimate cognitive status and affective and behavioral features. The cCT scans were performed primarily for routine clinical work-up and independently of the rest of the scientific study protocol in most cases. The median interval between CT imaging and biomaterial collection was 7 month, IQR 0–46 month. In 10 cases, CT was performed at the time of study inclusion (*n* = 45 of 60 probands with PBC). The ‘total calcification score’ (TCS) was determined by three independent investigators (HW, MM, SH) when a cCT was available.^2^ Ethylenediaminetetraacetic acid (EDTA) whole blood samples and serum were collected by peripheral venipuncture. Laboratory tests were performed to exclude secondary causes of brain calcifications by measuring serum calcium, phosphate, vitamin D, and parathyroid hormone levels. Calcium-phosphate metabolism was considered normal if the concentrations were within ±50% of the upper and lower reference limits.

### Clinical categorization

Participants were classified as symptomatic if they exhibited at least one of the following signs or symptoms that could not be attributed to a competing illness, based on the criteria adapted from Manyam *et al*.^13^ with minor modifications^13^: Parkinson’s syndrome, tremor, chorea, dystonia, spasticity, athetosis, orofacial dyskinesia, cerebellar syndrome, gait disorder, impaired fine motor skills, speech disorders, dysphagia, pyramidal symptoms, sensory impairment or pain, seizure, cognitive impairment including an abnormal age- and education-dependent MOCA or disinhibition, dysexecutive or memory deficits in the behavioral observation during anamnestic interview and neuropsychological assessment, symptoms leading to the diagnoses of a psychiatric disorder according to ICD-10 (the International Classification of Diseases, Tenth Revision), or a BDI-II of > 20 points, indicating the presence of at least moderate depression.^23^

Headache (22 out of 60; 36.67%) and specifically migraine (8 out of 60; 13.33%) were not regarded as sufficient to establish symptomatic PBC if headache was the only symptom, considering the high prevalence of headaches in the general population.

### Biosamples and laboratory tests

EDTA whole blood samples were collected by peripheral venipuncture. Plasma was collected after centrifugation of EDTA blood at 15°C for 2 × 10 min and at 15 000 × g. Biosamples were aliquoted to 300 µL samples and stored at −80°C. Samples were thawed only once directly before analysis.

Glial fibrillary acidic protein (GFAP) and phosphorylated tau (pTau) 181 were measured in serum by the Quanterix Simoa HD-1 analyzer using the Simoa GFAP Discovery Kit and Simoa pTau181 V2 Advantage Kit (Quanterix, MA, USA). Neurofilament light and heavy chains (NfL and NfH, respectively) were analyzed in serum using commercially available kits using the microfluidic ELLA system (Bio-Techne, Minneapolis, USA). Serum neuron-specific enolase (NSE) and S100 calcium-binding protein B (S100 B) were measured using an electrochemiluminescence immunoassay (ECLIA) on the Roche Cobas e411 (Roche, Basel, Switzerland). Calcium, phosphate, parathyroid hormone (PTH), and vitamin D were analyzed with Atellica Solution using the Atellica CH Analyzer and Atellica IM Analyzer (Siemens Healthineers, Tarrytown,USA). All measurements were conducted according to the manufacturer’s instructions. Patients and control samples were randomly distributed on the different assay plates. Serum quality controls were included in all runs. Intra- and inter-assay coefficients of variation were <15%.

### Genetic analysis

According to standard procedures, DNA was extracted from EDTA blood samples for genetic analysis. Whole exome and targeted Sanger sequencing were performed as described previously.^24^ The genetic testing results were not communicated according to the probands’ consent. When probands wanted to know their genetic status, they were referred to our routine clinical genetic test and counseling path.

### Statistical analysis

The statistical analysis was performed using IBM SPSS Statistics-v. 28 (2022, IBM Corp, Armonk, NY, USA). Due to small sample sizes in certain subgroups and non-normally distributed data, two-sided non-parametric statistical test were used including the Mann–Whitney U test or the global Kruskal-Wallis test followed by Dunn's post hoc test. Testing was in all cases two-sided. A *P*-value of <0.05 was considered statistically significant. Non-parametric partial correlations were determined using age as a co-variate of no interest.^25^

## Results

### Patient characteristics

Six hospitals recruited 60 probands with PBC to the German Fahr-NET register. The family history was unobtainable from two probands, while 12 out of 58 (20.69%) subjects with PBC (asymptomatic and symptomatic) reported imaging-confirmed calcifications in their relatives. Furthermore, 25 out of 58 (43.10%) reported a family history of symptoms compatible with P(F)BC. WES revealed a total of 22 different rare genetic variants in known PBC disease genes [*SLC20A2*, *n* = 7; *PDGFB*, *n* = 3; *PDGFRB*, *n* = 2; *XPR1*, *n* = 3; *MYORG*, *n* = 6 and *JAM2*, *n* = 1; (Supplementary Table 2)] in 28 individuals. Seven of these variants have been previously described in PBC, while 15 were novel. However, pathogenicity could not be definitively proven for all detected variants (Supplementary Table 2). Nevertheless, considering that all variants affected highly conserved amino acids and/or represented loss-of-function mutations in haploinsufficient genes and/or had been previously reported in PBC patients, the probands with genetic variants in P(F)BC genes were further referred to as ‘genetic’, while those without as ‘no identified mutation’. Two probands were excluded from comparisons of the groups ‘genetic’ versus ‘no identified mutation’ since one proband carried a heterozygous rare variant in *JAM2*, a recessive PBC gene and the other showed a ‘likely benign’ *XPR1* variant (cases 1 and 28; Supplementary Table 2).

Based on the deep phenotyping described above (see ‘clinical categorization’ in materials and methods), 60% (*n* = 36 out of 60) of the probands were symptomatic. The most frequently observed single symptom was mood disorder, followed by executive dysfunction (Table 1). Overall, we found that neuropsychological deficits among the patients were more frequent (*n* = 31; 51.67%) than purely neurological symptoms (*n* = 27; 45%), including movement disorders.

**Table 1:** Epidemiological data of all probands with PBC and further specified according to the genetic status

	All with PBC	Genetic PBC	No identified mutation PBC
**Cases, *n***	60	26	30
**Age at evaluation, median (IQR)**	56.30 (49.35–69.80)	55.50 (44.75–63.50)	64.00 (50.40–74.00)
**Sex (male/female)**	30/30	19/7	10/20
**Asymptomatic, *n***	20	2	16
Percentage (%)	33.33	7.69	53.33
Age at evaluation (median (IQR))	54.00 (39.45–69.58)	27.15	60.00 (45.00–73.98)
**Symptomatic, *n***	36	20	14
Percentage (%)	60.00	76.92	46.67
Age at evaluation (median (IQR))	60.80 (49.85–69.95)	58.10 (49.23–64.10)	64.10 (52.30–74.00)
**Missing clinical information, *n* (%)**	4 (6.67)	4 (15.38)	0 (0)
**Neurological disorders, *n* (%)**	27 (45)	18 (69.23)	7 (23.33)
Parkinsonism, *n* (%)	8 (13.33)	4 (15.38)	4 (13.33)
Tremor, *n* (%)	13 (21.67)	9 (34.62)	3 (10)
Chorea, *n* (%)	0 (0)	0 (0)	0 (0)
Dystonia, *n* (%)	2 (3.33)	2 (7.69)	0 (0)
Spasticity, *n* (%)	3 (8.33)	1 (3.85)	1 (3.33)
Athethosis, *n* (%)	0 (0)	0 (0)	0 (0)
Orofacial dyskinesia, *n* (%)	0 (0)	0 (0)	0 (0)
Speech disorder, *n* (%)	13 (21.67)	10 (38.46)	2 (6.67)
Dysphagia, *n* (%)	3 (8.33)	1 (3.85)	1 (3.33)
Cerebellar, *n* (%)	10 (16.67)	7 (26.92)	2 (6.67)
Pyramidal, *n* (%)	11 (18.33)	8 (30.77)	2 (6.67)
Gait disorder, *n* (%)	13 (21.67)	8 (30.77)	4 (13.33)
Sensory/Pain, *n* (%)	2 (3.33)	0 (0)	1 (3.33)
Fine motor skills, *n* (%)	15 (25)	10 (38.46)	4 (13.33)
Seizure, *n* (%)	1 (1.67)	1 (3.85)	0 (0)
**Neuropsychological deficits, *n* (%)**	31 (51.67)	17 (65.38)	12 (40)
Cognitive disorder overall, *n* (%)	20 (33.33)	13 (50.00)	6 (20)
Memory, *n* (%)	14 (23.33)	11 (42.31)	3 (10)
Dysexecutive, *n* (%)	16 (26.67)	11 (42.31)	4 (13.33)
Apathia, *n* (%)^a^	15 (25)	7 (26.92)	8 (26.67)
Disinhibition, *n* (%)	4 (6.67)	3 (11.54)	1 (3.33)
Attention, *n* (%)^a^	24 (40)	14 (53.85)	8 (26.67)
Psychiatric disorder overall, *n* (%)	23 (38.33)	11 (42.31)	11 (36.67)
Psychosis, *n* (%)	1 (1.67)	1 (3.85)	0 (0)
Mood disorder, *n* (%)	23 (38.33)	11 (42.31)	11 (36.67)
**Questionnaires**			
MOCA [*n*; median (IQR)]	46; 25.00 (22.00–27.25)	18; 24.00 (21.75–27.25)	24; 25.00 (24.00–26.75)
PDQ-39 [*n*; median (IQR)]	44; 14.06 (5.21–23.86)	18; 15.31 (5.40–24.13)	22; 14.63 (6.91–24.59)
UPDRS Part 3 [*n*; median (IQR)]	45; 2.00 (0.00–16.00)	19; 10.00 (0.00–19.00)	22; 2.00 (0.00–11.25)
UPDRS total [*n*; median (IQR)]	44; 14.50 (5.25–31.75)	18; 21.00 (6.75–33.00)	22; 13.00 (5.75–28.75)
Bathel Index [*n*; median (IQR)]	48; 100.00 (95.00–100.00)	20; 100.00 (95.00–100.00)	24; 100.00 (96.25–100.00)
SARA [*n*; median (IQR)]	44; 1.00 (0.00–4.875)	18; 1.50 (0.00–7.00)	22; 1.00 (0.00–3.13)

^a^Not decisive for the classification into symptomatic and asymptomatic.

### Correlation of calcification load and clinical deficits

**Figure 1:**
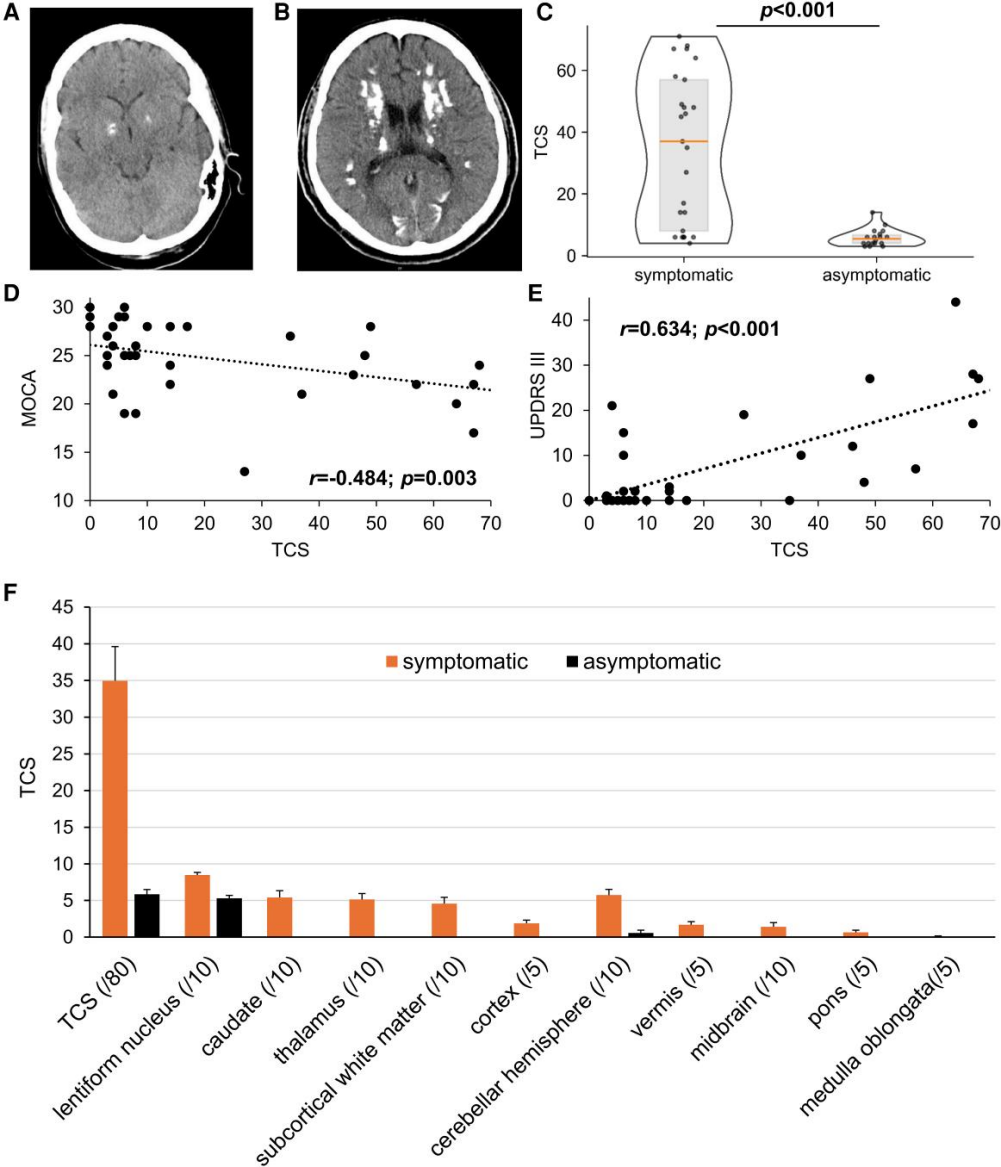
**Imaging cut-off threshold that indicates presence of symptoms in primary brain calcification.** Exemplary CT images with (**A**) mild calcifications restricted to the globus pallidus internus of an asymptomatic proband compared with (**B**) a symptomatic individual with primary brain calcification (PBC) with calcifications also in other brain regions. (**C**) Violin plot with median and interquartile range of the total calcification score (TCS) of symptomatic (*n* = 25) and asymptomatic subjects (*n* = 18) analysed by the Mann–Whitney U-test. Scatter plots in (**D**) and (**E**) demonstrate the correlation between TCS and cognition [Montreal Cognitive Assessment (MOCA) score; *r* = −0.484; *P* = 0.003; *n* = 35] as well as Parkinsonian motor symptoms (UPDRS III score; *r* = 0.634; *P* < 0.001; *n* = 35), respectively. The Spearman-Rho correlation was calculated. (**F**) Mean ± S.E.M. of calcification scores (TCS and subscores of TCS for the indicated anatomical brain regions) of symptomatic (*n* = 25) and asymptomatic (*n* = 18) patients. Lentiform nucleus = pallidum and putamen. *n* = number of probands.

The ‘total calcification score’ (TCS) is an established CT-based score to describe the overall or region-specific PBC calcification load.^2^ In this study, the TCS was determined by three independent assessors in patients for whom a cCT was available from previous routine clinical workups (*n* = 45 of 60 with PBC). Total TCS ranged from 3 to 71 out of a maximum possible score of 80 (*median* 8.00, *IQR* = 6.00–45.50; Fig. 1A–C). Individuals with symptomatic PBC had a significantly higher total calcification score when compared with PBC probands without symptoms (Table 2 and Fig. 1C). Moreover, the TCS correlated significantly with severity of several clinical scores, specifically MOCA, SARA, Barthel Index, and UPDRS part III as well as UPDRS I-III (Fig. 1D and E and Supplementary Table 3).

**Table 2:** Overview of the statistical results of the mean value comparisons of the TCS analyzed using the Mann–Whitney U-test and the non-parametric partial correlations

Mean value comparisons									
Biomarker		symptomatic PBC	asymptomatic PBC	genetic PBC	no identified mutation PBC	genetic PBC except *MYORG*	*MYORG* mutation carriers	*SLC20A2* mutation carriers	*P*-value
**TCS**	Median (IQR); *n*	37.00 (7.00–57.50); 25	5.50 (4.00–7.25); 18						**<0**.**001**
	Median (IQR); *n*			41.00 (14.00–60.25); 18	6.00 (4.00–8.00); 23				**<0**.**001**
	Median (IQR); *n*					31.00 (12.00–46.50); 14	67.50 (67.00–70.25); 4		**<0**.**001**
	Median (IQR); *n*						67.50 (67.00–70.25); 4	35.00 (23.00–46.00); 7	**0**.**006**

Correlations			
Calcium			
Questionnaire	*r*-value	*P*-value	*n*
MOCA	0.323	**0**.**027**	47
UPDRS I-III	−0.361	**0**.**014**	46

NfL			
Questionnaire	*r*-value	*P*-value	*n*
TCS	0.410	**0**.**009**	43
MOCA	−0.363	**0**.**014**	46
UPDRS III	0.428	**0**.**003**	46
UPDRS I-III	0.428	**0**.**003**	45
SARA	0.447	**0**.**002**	45
Barthel Index	−0.496	**<0**.**001**	47

GFAP			
Questionnaire	*r*-value	*P*-value	*n*
TCS	0.241	0.166	38

NfH			
Questionnaire	*r*-value	*P*-value	*n*
TCS	0.086	0.587	43

Statistically significant values are highlighted in bold.

A region-specific analysis showed that all PBC probands displayed calcifications in the pallidum (Fig. 1F). In symptomatic probands with PBC, at least one region beyond the pallidum was involved. In contrast, asymptomatic calcifications were almost always restricted to the pallidum. There were only two exceptions of asymptomatic PBC probands with minor calcifications in the cerebellar hemispheres in addition to pallidal calcification (Fig. 1F).

**Figure 2:**
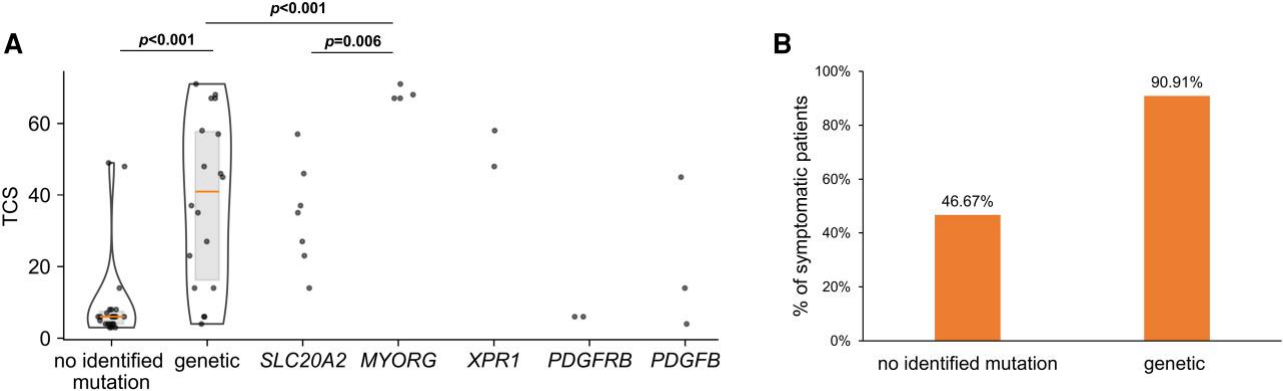
**Impact of a mutation on the severity of calcifications and frequency of symptoms.** (**A**) Violin plot with median and interquartile range of the total calcification scores (TCS) comparing probands with primary brain calcification (PBC) with no identified genetic variant (*n* = 23) and with a likely causative genetic variant (*n* = 18) using the Mann–Whitney U-test. The ‘genetic’-group was further grouped by the specific mutated disease genes *SLC20A2* (*n* = 7), *MYORG* (*n* = 4), *XPR1* (*n* = 2), *PDGFRB* (*n* = 2) and *PDGFB* (*n* = 3) and also analyzed using the Mann–Whitney U test. (**B**) Proportion of symptomatic subjects in the respective groups of no identified mutation (*n* = 14 of 30) and genetic PBC (*n* = 20 of 22). *n* = number of probands.

Probands with genetic PBC had a significantly higher calcification load than probands without an identified mutation in a known PBC gene (Table 2 and Fig. 2A). We also observed a significantly higher TCS in probands with a *MYORG* variant compared with the TCS of all other people with genetic PBC and *SLC20A2* mutation carriers (Table 2 and Fig. 2A).

The vast majority of genetic PBC probands were symptomatic (Fig. 2B). However, one very young (18 years old) proband with the *SLC20A2* mutation c.1523 + 1G > T, displayed calcifications, which were restricted to the pallidum (Supplementary Table 2) but did not present neurological or neuropsychological symptoms. Moreover, pallidal calcifications were accidentally detected by a CT scan in a 27-year-old female patient admitted to the stroke unit with suspected transitory ischemic attack. Subsequent WES revealed a rare genetic variant in *PDGFRB*. (Supplementary Table 2).

### Genotype-dependent alteration of parathyroid hormone levels

Five out of 63 probands had calcium, phosphate, or PTH values more than 50% outside the upper or lower reference limit. One had elevated PTH level (305.8 ng/L; reference range 18.5–88 ng/L), low calcium levels (1.74 mmol/L; reference range 2.18–2.60 mmol/L), and normal phosphate levels (1.26 mmol/L; reference range 0.84–1.45 mmol/L) in line with secondary hyperparathyroidism, while another two displayed PTH levels below the measurable level (<4.6 ng/L) in combination with low serum calcium (1.78 mmol/L and 1.91 mmol/) and high phosphate (2.17 mmol/L and 1.71 mmol/L) levels, suggesting primary hypoparathyroidism. Due to a probable secondary cause of brain calcification, these three probands were not included in the study resulting in a sample size of 60 probands that we further analysed. The other two related patients showed normal calcium concentrations (2.56 mmol/L and 2.44 mmol/L), mildly lowered parathyroid hormone concentrations (12.3 ng/L and 11.2 ng/L), and elevated phosphate concentrations (3.26 mmol/L and 2.87 mmol/L), suggesting familial hyperphosphatemia. Because of the mild alterations in PTH levels and normal calcium levels as well as rare, likely pathogenic and homozygous *MYORG* mutations (cases 2 and 3; Supplementary Table 2) these two probands were further analyzed, resulting in a finally analyzed cohort of 60 probands with PBC. Individuals with PBC had values for PTH [44.20 ng/l (*IQR* 26.50–59.70)], calcium [2.35 mmol/l (*IQR* 2.30–2.40)] and phosphate [1.16 mmol/l (*IQR* 1.04–1.25)] within ±50% of the upper and lower reference limit including all other *MYORG* mutation carriers.

**Figure 3:**
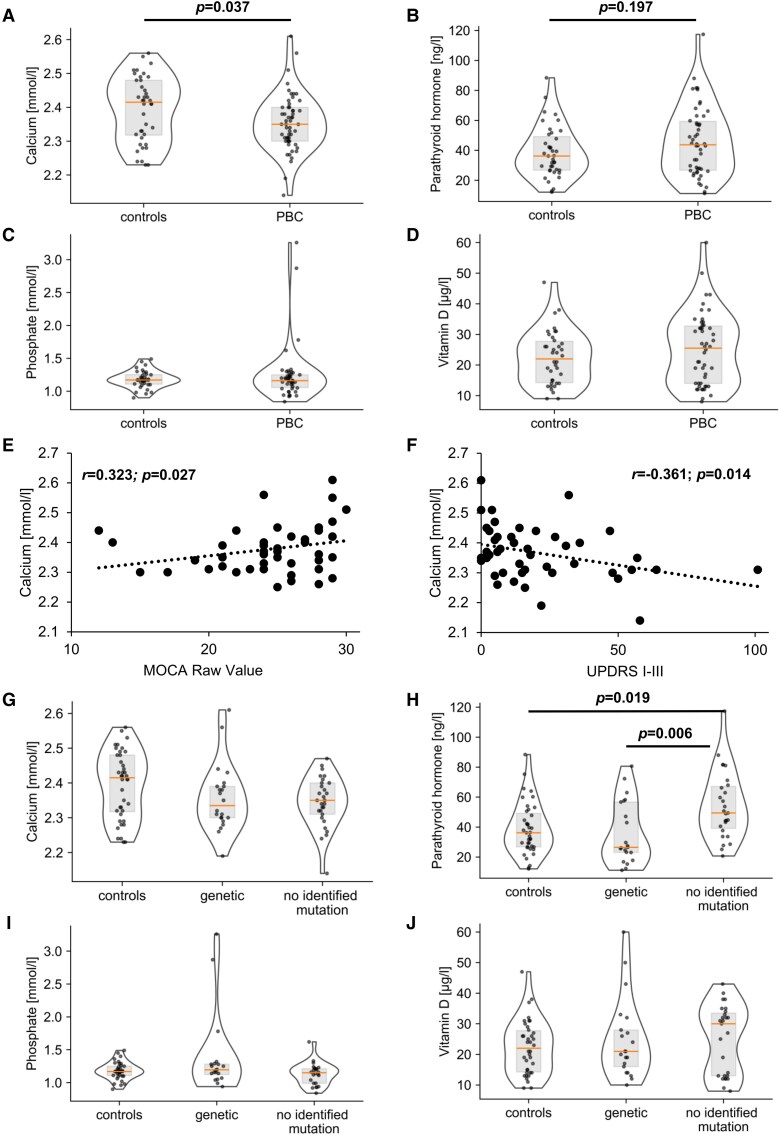
**Calcium metabolism in patients with primary brain calcification.** (**A-D**) Violin plots with median and interquartile range comparing controls and all probands with primary brain calcification (PBC) regarding (**A**) serum calcium levels (*n PBC* = 55, *n controls* = 40), (**B**) serum parathyroid hormone (*n PBC* = 50, *n controls* = 39), (**C**) serum phosphate (*n PBC* = 46, *n controls* = 36) and (**D**) serum vitamin D (*n PBC* = 50, *n controls*  *=* 38) analyzed by the Mann–Whitney U-test. (**E, F**) Scatter plot with trend line of the Spearman-Rho correlation between (**E**) cognition (Montreal Cognitive Assessment (MOCA) score; *r* = 0.323; *P* = 0.027; *n* = 47) and (**F**) Parkinsonian motor symptoms (UPDRS I-III score; *r* = −0.361; *P* = 0.014; *n* = 46) with calcium. (**G–J**) Violin plots with median and interquartile range comparing control probands, genetic PBC and PBC with no identified mutation using the global Kruskal-Wallis test followed by Dunn's post hoc test for (**G**) serum calcium (*n genetic PBC* = 22, *n no identified mutation PBC* = 29, *n controls* = 40), (**H**) serum parathyroid hormone (*n genetic PBC* = 21, *n no identified mutation PBC* = 27, *n controls* = 39), (**I**) serum phosphate (*n genetic PBC* = 20, *n no identified mutation PBC* = 24, *n controls* = 36) and (**J**) and serum vitamin D (*n genetic PBC* = 21, *n no identified mutation PBC* = 27, *n controls* = 38). *n* = number of probands.

However, a mild decrease in serum calcium levels within the normal range was observed in probands with PBC compared with age- and sex-matched controls (Table 3 and Fig. 3A; for details of control probands see Supplementary Table 1 and 4). On the other hand, a non-significant trend towards increased PTH levels (Table 3 and Fig. 3B) and unaltered phosphate and vitamin D levels were observed (Fig. 3C and D). Calcium concentrations showed a weak positive association with cognitive performance measured by the MOCA score (Table 2 and Fig. 3E) and a negative association with UPDRS I-III (Table 2 and Fig. 3F).

**Table 3:** Overview of the statistical results of mean value comparisons analyzed using the Mann–Whitney U-test or the global Kruskal-Wallis test followed by Dunn's post hoc test

Biomarker		controls	all PBC	symptomatic PBC	asymptomatic PBC	genetic PBC	no identified mutation PBC	*P*-value
**Calcium**	Median (IQR); *n*	2.42 mmol/L (2.31–2.48); 40	2.35 mmol/L (2.30–2.40); 55					**0**.**037**
	Median (IQR); *n*	2.42 mmol/L (2.31–2.48); 40					2.35 mmol/L (2.31–2.40); 29	0.15
**PTH**	Median (IQR); *n*	36.20 ng/L (26.70–50.60); 39	43.70 ng/L (26.40–60.50); 50					0.197
	Median (IQR); *n*					26.50 ng/L (20.40–57.10); 21	49.40 ng/L (37.70–68.00); 27	**0**.**006**
	Median (IQR); *n*	36.20 ng/L (26.70–50.60); 39					49.40 ng/L (37.70–68.00); 27	**0**.**019**
**pTau181**	Median (IQR); *n*	13.45 pg/mL (8.62–17.00); 20	13.14 pg/mL (9.63–17.80); 35					0.681
**NSE**	Median (IQR); *n*	14.60 µg/L (13.10–16.65); 29	15.40 µg/L (12.50–20.45); 37					0.310
**S100**	Median (IQR); *n*	0.05 µg/L (0.04–0.08); 39	0.07 µg/L (0.05–0.08); 45					0.278
**GFAP**	Median (IQR); *n*	115.00 pg/mL (95.00–150.00); 39	171.50 pg/mL (100.80–235.18); 44					**0**.**014**
	Median (IQR); *n*	115.00 pg/mL (95.00–150.00); 39		190.31 pg/mL (123.07–225.53); 22				**0**.**012**
	Median (IQR); *n*	115.00 pg/mL (95.00–150.00); 39			138.56 pg/mL (72.50–319.02); 20			0.665
	Median (IQR); *n*	115.00 pg/mL (95.00–150.00); 39				190.31 pg/mL (89.43–210.25); 18	143.06 pg/mL (102.25–256.18); 24	0.091
**NfL**	Median (IQR); *n*	16.00 pg/mL (11.25–21.00); 40	21.00 pg/mL (13.50–37.00); 53					**0**.**013**
	Median (IQR); *n*	16.00 pg/mL (11.25–21.00); 40		26.00 pg/mL (15.00–45.00); 31				**0**.**008**
	Median (IQR); *n*	16.00 pg/mL (11.25–21.00); 40			21.00 pg/mL (13.00–24.00); 20			1.000
	Median (IQR); *n*	16.00 pg/mL (11.25–21.00); 40				23.50 pg/mL (13.50–40.25); 20	21.00 pg/ml (14.00–34.00); 29	**0**.**034**
	Median (IQR); *n*	16.00 pg/mL (11.25–21.00); 40				23.50 pg/mL (13.50–40.25); 20		0.128
	Median (IQR); *n*	16.00 pg/mL (11.25–21.00); 40					21.00 pg/mL (14.00–34.00); 29	0.071
**NfH**	Median (IQR); *n*	186.50 pg/mL (85.25–314.50); 40	259.00 pg/mL (65.00–584.50); 53					0.12
	Median (IQR); *n*	186.50 pg/mL (85.25–314.50); 40		273.00 pg/mL (61.00–603.00); 31				0.304

Statistically significant values are highlighted in bold.

When considering the genetic status, vitamin D and phosphate levels (Fig. 3I and J) were not significantly altered between genetic or no identified mutation PBC compared with controls. However, a significantly increased PTH level distinguished PBC individuals with no identified genetic cause from genetic cases or controls (Table 3 and Fig. 3H). Moreover, there was also a trend towards lower serum calcium in no identified mutation PBC when compared with controls, however it did not reach statistical significance (Table 3 and Fig. 3G). The results point to a mild alteration of calcium metabolism, most pronounced in no identified mutation PBC.

### Elevated serum GFAP and neurofilament light chain levels in PBC

Since CSF analysis is not part of the routine workup of probands with PBC, we measured established serum biomarkers for neurodegenerative diseases. Specifically, levels of NfH and NfL, pTau181, and NSE linked to neuronal pathology, as well as GFAP and S100B protein, which are indicators of chronic or more acute astrocytosis, respectively, were measured.

pTau is a marker of an often amyloid-and/or neuronal activity-related change in tau phosphorylation. Compared with controls, serum pTau and NSE did not change in probands with PBC (Table 3). NSE values also represent a quality measure since hemolysis increases serum NSE values.

**Figure 4:**
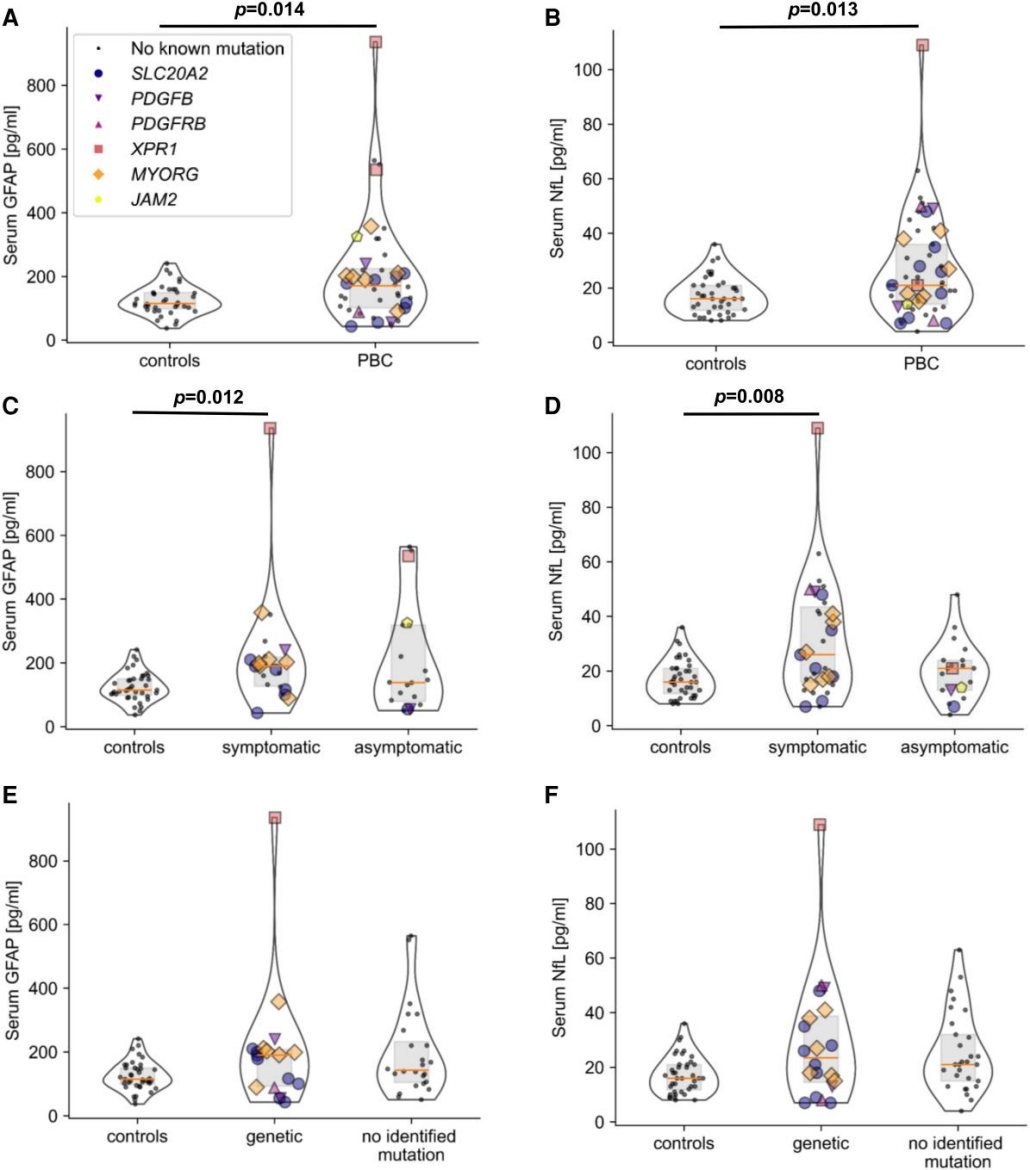
**GFAP and neurofilament serum levels in probands with primary brain calcification.** (**A, B**) Violin Plots with median and interquartile range comparing controls and probands with primary brain calcification (PBC) using the Mann–Whitney U-test for (**A**) serum glial fibrillary acidic protein (GFAP) (*n controls* = 39, *n PBC* = 44) and (**B**) neurofilament light chain (NfL) (*n controls* = 40, *n PBC* = 53). (**C, D**) Violin Plots with median and interquartile range comparing controls, symptomatic and asymptomatic probands with PBC using the global Kruskal-Wallis test followed by Dunn's post hoc test for (**C**) GFAP (*n controls* = 39, *n symptomatic* = 22, *n asymptomatic* = 20) and (**D**) NfL (*n controls* = 40, *n symptomatic* = 31, *n asymptomatic* = 20). (**E, F**) Violin Plots with median and interquartile range comparing controls, genetic and no identified mutation probands with PBC using the global Kruskal-Wallis test followed by Dunn's post hoc test for (**E**) GFAP (*n controls* = 39, *n genetic* = 18, *n no identified mutation* = 24) and (**F**) NfL (*n controls* = 40, *n genetic* = 20, *n no identified mutation* = 29). *n* = number of probands.

Also S100B protein levels remained unchanged between PBC and control condition (Table 3). S100B is regarded as a marker for astrocyte alterations. However, its expression is more widespread and typically elevated in CSF and blood upon acute damage conditions such as ischemic stroke or cerebral hemorrhage, which unselectively affect all brain cell types.^26,27^ In contrast, GFAP is more specifically expressed in a subset of astrocytes and increases in chronic conditions with astrocytosis, such as amyotrophic lateral sclerosis (ALS).^28,29^ In contrast to S100B, we found GFAP serum levels to be significantly increased in PBC probands when compared with control individuals (Table 3 and Fig. 4A).

Moreover, while only a trend to higher serum NfH concentrations in probands with PBC was observed, serum NfL values were significantly increased in probands with PBC (Table 3 and Fig. 4B).

In a separate analysis of symptomatic and asymptomatic probands with PBC, both GFAP and NfL, but not NfH concentrations were significantly higher in the subset of symptomatic probands with PBC compared with controls (Table 3 and Fig. 4C and D). Probands with PBC who did not reveal symptoms upon deep phenotyping, however, did not reveal increased GFAP or NfL levels in serum (Table 3 and Fig. 4C and D). In agreement with previous data, both GFAP and neurofilaments correlated with the age of probands (Supplementary Fig. 1).^30-32^ However, age was not a confounder when comparing PBC and controls, and both groups matched concerning age and sex (*P-value controls* versus *PBC* = 0.673; *P-value asymptomatic* versus *symptomatic* versus *controls* = 0.687; *P-value no identified mutation* versus *genetic* versus *controls* = 0.413; Supplementary Table 4).

No significant biomarker alteration was observed between PBC probands with a genetic variant in known PBC genes compared with individuals with no identified mutation (Fig. 4E and F). GFAP showed a strong trend towards elevated concentrations in both genetic and no identified mutation PBC compared with controls but did not reach statistical significance (Table 3 and Fig. 4E). NfL levels reached statistical significance when analyzed with the global Kruskal-Wallis test. However, a further group analysis with the Dunn's post hoc test did not (Table 3 and Fig. 4F). No genotype-dependent difference was noted for NfH levels compared with controls.

### Correlation of biomarkers with clinical and imaging scores

**Figure 5:**
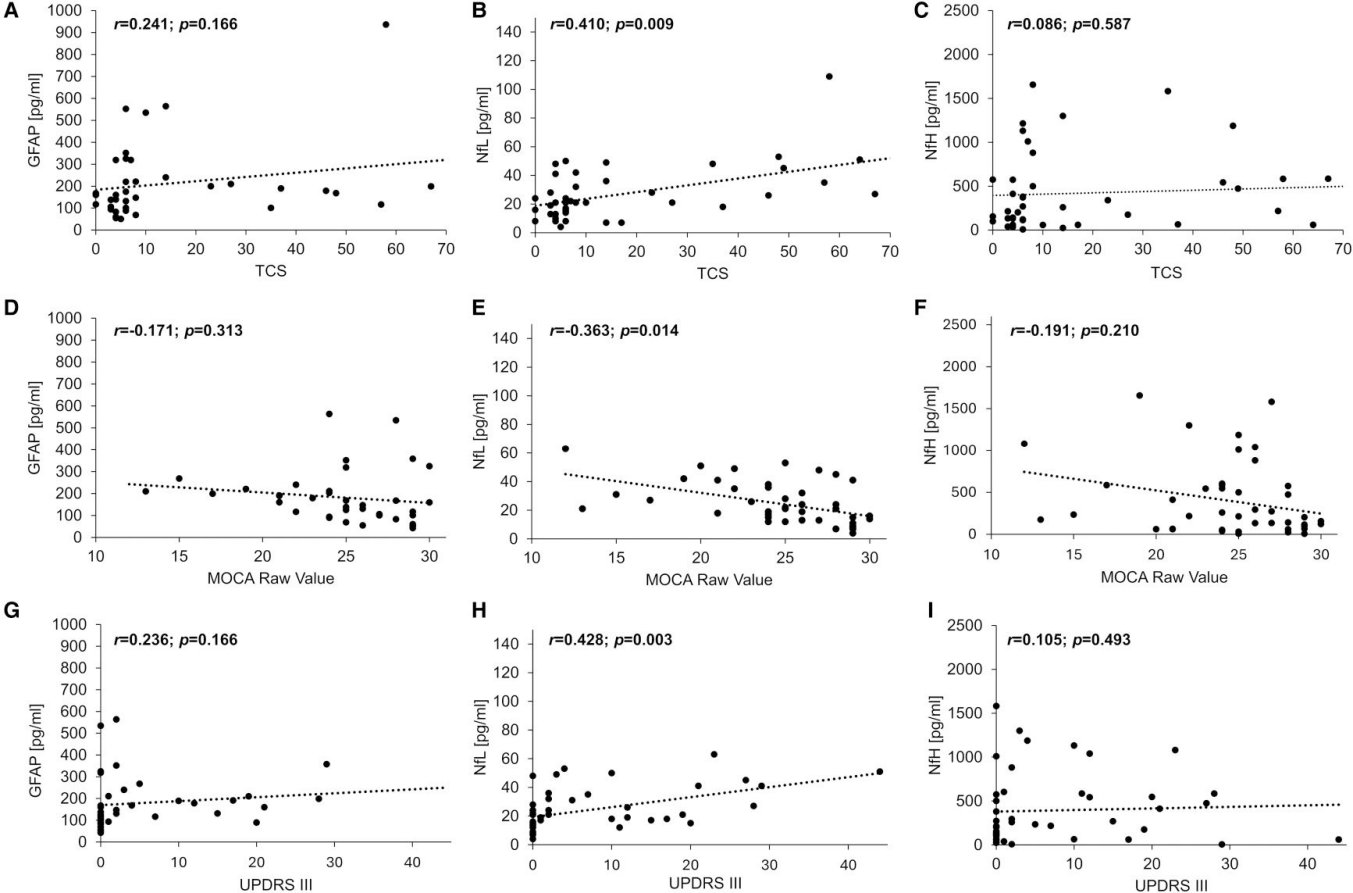
**Spearman-rho correlations of serum biomarkers with calcifications, cognition and parkinsonian symptoms.** (**A–C**) Correlation between (**A**) serum glial fibrillary acidic protein (GFAP) (*r* = 0.241; *P* = 0.166, *n* = 38), (**B**) serum neurofilament light chain (NfL) (*r* = 0.410; *P* = 0.009; *n* = 43) and (**C**) serum neurofilament heavy chain (NfH) (*r* = 0.086; *P* = 0.587; *n* = 43) with the extend of the brain calcifications [total calcifications score (TCS)]. (**D–F**) Correlation between (**D**) serum GFAP (*r* = −0.171; *P* = 0.313; *n* = 38), (**E**) serum NfL (*r* = −0.363; *P* = 0.014; *n* = 46) and (**F**) serum NfH (*r* = −0.191; *P* = 0.210; *n* = 46) with the Montreal Cognitive Assessment (MOCA) score. (**G–I**) Correlation between (**G**) serum GFAP (*r* = 0.236; *P* = 0.166; *n* = 37), (**H**) serum NfL (*r* = 0.428; *P* = 0.003; *n* = 46) and (**I**) serum NfH (*r* = 0.105; *P* = 0.493; *n* = 46) with the UPDRS III score for Parkinsonian motor deficits. *n* = number of probands.

We assessed whether serum GFAP, NfL or NfH are associated with the calcification load or clinical scores, considering the significant alterations of GFAP and NfL serum concentrations in symptomatic but not asymptomatic probands with PBC. We found that NfL but not NfH or GFAP correlated with the TCS (Table 2 and Fig. 5A–C). Moreover, NfL serum concentrations correlated significantly with the MOCA score (Fig. 5E), the UPDRS III (Fig. 5H), the sum of UPDRS I-III (not shown in the figure) and the SARA score as well as the Barthel Index (Table 2). Thus, we found evidence for a significant association between serum NfL concentrations and measures for calcification load and neurological and cognitive impairment.

## Discussion

We present comprehensive analyses integrating clinical and imaging phenotyping, genotyping, and serum biomarker profiling in individuals with PBC. Our study offers evidence of glial and neuronal alterations, establishing associations among brain calcifications, clinical deficits, genetics, and blood biomarkers. Furthermore, our findings highlight a genotype-dependent variation in parathyroid hormone levels.

Golüke *et al*.^33^ also observed an association between brain calcification and clinical severity. Similarly, we could also show that the calcification load is significantly associated with clinical deficit scores, specifically the neuropsychological MOCA score, movement disorders (UPDRS III and I-III, SARA), and reduced daily living activities (Barthel Index). Our data also confirmed previous results showing that patients with a genetic cause for PBC have both a higher and more extensive degree of calcification and are more likely to experience PBC-related symptoms.^33^ Furthermore, we demonstrate that calcifications extending the pallidum are most likely associated with clinical symptoms, providing a practically relevant imaging cut-off indicating the clinical manifestation of PBC.

The detailed phenotyping of 60 subjects with PBC revealed a broad spectrum of clinical syndromes. We found that neuropsychological deficits are more frequent than neurological deficits, demonstrating that the view of PBC predominantly as an atypical movement disorder or Parkinson’s syndrome is not justified. This broad phenotypic spectrum and lack of a defined clinical syndrome likely contribute to the lack of consistent recognition of PBC as a distinct clinical entity. A crucial future question will be which factors ultimately lead to a specific set of symptoms.

The observation that pallidal calcifications were revealed in all cases described here suggests that the calcified lesions start focally in the pallidum, with subsequent pathology extending to other brain areas as the disease progresses. Additional minor calcifications in the cerebellar hemispheres found in only two asymptomatic probands and more pronounced cerebellar calcifications in all severely affected probands suggest that the cerebellum is possibly the second most susceptible region. Consequently, while it is crucial to refrain from automatically diagnosing and communicating mild primary brain calcifications to patients as a disease, it is worth noting that some individuals with a low calcification burden confined to the pallidum may be in a pre-symptomatic stage.^1,34^ This view is mainly supported by young mutation carriers with restricted pallidal pathology at the time of examination. (this study and^5,35^) Thus, although longitudinal data on imaging findings are scarce and clinical follow-up data are missing, currently available data suggest a slow, insidious onset of PBC symptoms over years or even decades.^13^ To avoid a substantial diagnostic delay, younger subjects with PBC should be followed up at least in longer intervals, even if asymptomatic, with a particular emphasis on neuropsychological examination.

Few PBC autopsy cases have been reported.^21,22,36^ We tested CNS-related laboratory biomarkers to better understand possible cellular pathology and its relationship with clinical symptoms. Concerning future therapeutic studies, laboratory surrogates indicating response to therapy seem all the more relevant, considering that PBC may progress very slowly and changes in disease activity are difficult to detect using clinical outcomes.

We investigated serum biomarkers since a lumbar puncture is usually not part of the clinical workup of PBC, and CSF is, therefore, rarely available. Neurofilaments (neurofilament light and heavy chain: NfL and NfH, respectively), which are strongly increased in ALS but are also elevated in other neurodegenerative diseases such as atypical Parkinson’s syndromes, are suggested to indicate neuronal damage.^37^ NfL was significantly elevated in PBC probands compared with controls. In contrast, the PBC group's tendency towards increased NfH serum levels did not reach statistical significance. Similarly, in other neurodegenerative diseases such as ALS, serum NfL has a higher diagnostic potency than serum NfH.^38^

The elevation of serum GFAP indicates astrocyte activation in PBC. While GFAP is regarded as a marker of more chronic astrocyte activation, S100B is also expressed by other glial cells and is increased in several acute lesion situations, often accompanied by an impairment of the BBB.^39,40^ In contrast to GFAP, serum S100B remained unaltered, further arguing for a more chronic, long-term activation of glial cells in PBC and a slow progression of PBC pathology over years or even decades with minor neurovascular unit impairment. This hypothesis aligns with the non-significant nominal trend to elevated neurofilament and GFAP serum levels observed in asymptomatic individuals with PBC.

We observed a significant correlation of NfL serum levels with the calcification load and several clinical scores for disease severity, such as the MOCA and the UPDRS score, the SARA ataxia score, and the Barthel Index. These findings underscore the relevance of serum biomarkers for PBC as potential surrogates for disease severity.

The mechanistic relevance of astrocytic activation for PBC is underscored by the expression of PBC-causing genes predominantly in cell types forming the neurovascular unit.^41^ The PBC gene *MYORG* is even predominantly expressed in astrocytes.^12^ Nevertheless, we observed the highest GFAP serum values in our cohort in the two PBC patients with mutations in the PBC gene *XPR1* (534.93 pg/mL and 936.1 pg/mL). It remains to be demonstrated whether *XPR1* mutant patients have an exceptionally high degree of astrocytosis when CNS tissue from autopsies becomes available.

The diagnosis of PBC implies that a likely secondary cause of calcifications was excluded.^1^ From our initial cohort, three subjects had to be excluded from further analysis due to either severely increased or undetectable PTH levels, implying hyper- or hypoparathyroidism. However, although formally within the reference range, serum calcium levels turned out to be mildly but significantly reduced in the remaining PBC cohort compared with controls. Moreover, PTH levels were significantly higher in no identified mutation PBC than in cases with a likely causative genetic variant or controls. This constellation principally agrees with mild and partially compensated pseudohypoparathyroidism in PBC with no identified mutation but not in genetic PBC.^42^ Without genetic causes, mild alterations of calcium regulation, such as a slightly reduced PTH sensitivity in target cells, could contribute to PBC in the long run. Modifying the calcium metabolism could, therefore, be considered a therapeutic strategy, particularly for no identified mutation PBC.

The main strength of our study is the fact it represents the first systematic assessment of established neurological biomarkers in a comparably high number of both asymptomatic people with PBC and patients with clinical manifestation of this rare disease, and the integration of multimodal data types, specifically clinical phenotyping data, next generation sequencing results, CT imaging as well as laboratory biomarkers. A limitation is that we present a cross-sectional study since longitudinal data are currently unavailable.

In conclusion, the study demonstrated a link between calcifications and quantitative clinical scales of neurological and neuropsychological deficits, pronounced in genetic cases. We provide a clinically relevant imaging pattern suggesting the likely presence of symptoms due to PBC in a clinical context. Moreover, serum biomarkers point to neuronal damage and astrocytic activation in symptomatic PBC, with a nominal but insignificant increase already in asymptomatic PBC. Thus, mild asymptomatic calcifications may also be premanifest and closer to disease than previously assumed.^43^ Consequently, these individuals, especially young or middle-aged, should be counseled and followed up, although our current insight suggests a rather slowly progressing disease over decades. Our biomarker findings are also relevant for a future therapeutic study design. The abnormalities in calcium regulation seen in no identified mutation PBC may pave the way to such a therapeutic approach.

## Supplementary Material

fcaf388_Supplementary_Data

## Data Availability

WES data are available upon request from J.H.W. The analyses were performed using standard statistics programs (i.e. SPSS). No additional, proprietary code has been used for this study.
