# Supplementary material for: Deep clinical, genetic, and serum biomarker profiling indicates glial and neuronal pathology in primary brain calcification
Source: Brain Commun. 2025 Oct 7;7(6):fcaf388. doi: 10.1093/braincomms/fcaf388 (PMC12574706; doi:10.1093/braincomms/fcaf388)

Supplementary information

“Deep clinical, genetic, and serum biomarker profiling indicates glial and neuronal pathology in primary brain calcification“

Schwahn et al.

Supplementary Table 1 Disease controls

Control	Info	Sex	Age	Recruiting center
1	Degeneration of the cervical spine	male	66	Halle, UMH
2	Trigeminal neuralgia	female	49	Halle, UMH
3	Functional	female	78	Halle, UMH
4	Myasthenia gravis	male	69	Halle, UMH
5	Headache	female	86	Halle, UMH
6	Dyplopia unexplained genesis	female	47	Halle, UMH
7	Dizziness of unknown etiology	female	41	Halle, UMH
8	Dizziness	male	45	Halle, UMH
9	Functional	male	61	Halle, UMH
10	Myalgias	male	55	Halle, UMH
11	Abducens palsy	female	78	Halle, UMH
12	Focal dysthonia	female	69	Halle, UMH
13	Headache	female	51	Halle, UMH
14	Benign fasciculations	female	52	Mannheim, UMM
15	Migraine	female	29	Mannheim, UMM
16	Benign fasciculations	male	53	Mannheim, UMM
17	Dysesthesias	female	35	Mannheim, UMM

Supplementary Table 2 Overview of the genetics of patients with PBC

**Sex**	**Patient**	**Age**	**Gene**	**Genomic Location**	**Variant cDNA**	**Variant Protein**	**GnomAD frequency (counts alternative allele homoz./heteroz./major allele homoz.)**	**Zygosity**	**Reported befor in ClinVar**
female	1	53	*JAM2*	chr21:27071114	c.520C>T	Arg174Cys	0/17/1613982	het	No
male	2,3	50 (P2), 40 (P3)	*MYORG*	chr9:34372752	c.190C>G	Gly64Arg	0/1/628372	hom	no
female	4	66	*MYORG*	chr9:34371335	c.1607C>T	Pro536Leu	0/1/1460510	hom	yes
male	5	58	*MYORG*	chr9:34371215	c.1727G>A	Arg576His	0/5/1458140	hom	no
female	6	61	*MYORG*	chr9: 34372603 - 34372614	c.329_340dup	Val110_Leu113dup	0/27/1604302	compound heterozygous	no
female	6	61	*MYORG*	chr9: 34371308	c.1634G>A	Gly545Asp	0/315/1611924	compound heterozygous	no
male, male, male	7,8,9	46, 49, 52	*MYORG*	chr9:34370975	c.1967T>C	Ile656Thr	1/105/1613446	hom	yes
male	10	81	*PDGFB*	deletion of at least the entire coding region	deletion	deletion	not in GnomAD	het	No
female	11	36	*PDGFB*	chr22:39629494	c.151G>A	Arg66Cys	0/8/1595914	het	No
male	12	84	*PDGFB*	chr22:39627638	c.445C>T	Arg149*	not in GnomAD	het	yes
female	13	27	*PDGFRB*	chr5:149500554	c.2483C>T	Ala828Val	0/1/832796	het	no
male	14	67	*PDGFRB*	chr5:149499076	c.2752T>C	Lys918Glu	0/1/832404	het	No
male	15	73	*SLC20A2*	chr8:42329800	c.109C>T	Val37Met	0/3/1461886	het	No
male	16,17,18	51, 56, 61	*SLC20A2*	chr8:42323381	c.344C>T	Thr115Met	0/4/780640	het	yes
male	19	56	*SLC20A2*	chr8:42317446	c.581A>G	Asn194Ser	0/18/1613350	het	No
male	20,21,22	61, 29, 20	*SLC20A2*	chr8:42294506	c.1523+1G>T		not in GnomAD	het	No
female	23	40	*SLC20A2*	chr8:42275478	c.1802C>T	Ser601Leu	0/26/1461046	het	yes
male	24	64	*SLC20A2*	chr8:42286347	c.1723G>A	Glu575Lys	0/2/1448922	het	yes
male	25	76	*SLC20A2*	chr8:42286272-42286274	c.1794+2_1794+4delTAG	-	not in GnomAD	het	No
male	26	57	*XPR1*	chr1: 180775250	c.500T>C	Leu167Pro	not in GnomAD	hom	No
female	27	64	*XPR1*	chr1:180775202	c.452T>C	Leu151Pro	not in GnomAD	het	No
	**Likely benign:**								
female	28	69	*XPR1*	chr1:180842993	c.1723A>G	Ile575Val	2/1033/1614158	het	yes

Supplementary Table 3 Association of clinical scores with the TCS

**Score**	***r-value***	***p-value***	***n***
MOCA	-0.484	0.003	35
SARA	0.541	<0.001	35
Barthel-Index	-0.427	0.007	38
UPDRS III	0.634	<0.001	35
UPDRS I-III	0.475	0.004	35

Supplementary Table 4 Age and gender matching of the groups

**Values**	**controls**	**all with PBC**	**asymptomatic/ presymptomatic**	**symptomatic**	**genetic**	**non-genetic**
mean (years)	55.94	56.75	54.53	58.65	54.07	58.81
median (years)	56.50	56.20	54.00	60.35	55.55	63.20
gender ratio (M/F)	17/24	30/30	6/14	21/15	19/7	10/20

Suppl. Figure 1 Correlations of serum biomarkers with age.

Scatter plot with trend line of linear associations between age and (A) glial fibrillary acidic protein (GFAP) (r=0.528; p<0.001; *PBC cases n*=44, *controls n*=39; *both groups pooled n*=83), (B) neurofilament light chain (NfL) (r=0.579; p<0.001; *PBC cases n*=53, *controls n*=40; *pooled n*=93) and (C) neurofilament heavy chain (NfH) (r=0.336; p<0.001; *PBC cases n*=53, *controls n*=40, *pooled n*=93). Blue dots: PBC cases, orange dots: controls. PBC and control probands were pooled and analyzed as one group using the Spearman-Rho correlation.

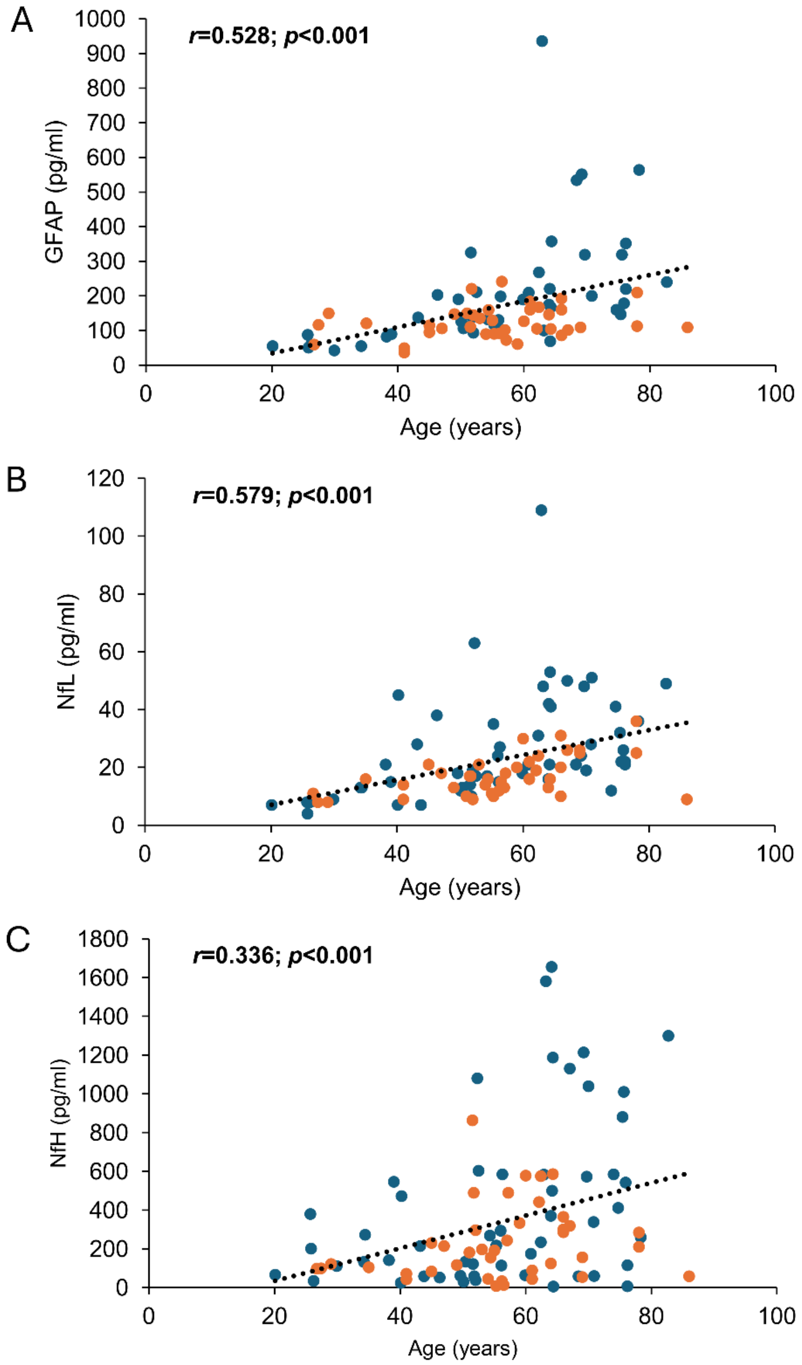

Supplement: fcaf388_Supplementary_Data [file fcaf388_supplementary_data.pdf]
